# External Validation of the IMPROD-MRI Volumetric Model to Predict the Utility of Systematic Biopsies at the Time of Targeted Biopsy

**DOI:** 10.3390/jcm12175748

**Published:** 2023-09-04

**Authors:** Antonella Ninivaggi, Francesco Guzzi, Alessio Degennaro, Anna Ricapito, Carlo Bettocchi, Gian Maria Busetto, Francesca Sanguedolce, Paola Milillo, Oscar Selvaggio, Luigi Cormio, Giuseppe Carrieri, Ugo Giovanni Falagario

**Affiliations:** 1 Department of Urology and Organ Transplantation, University of Foggia, 71122 Foggia, Italyugofalagario@gmail.com (U.G.F.); 2 Department of Urology, Bonomo Teaching Hospital, 76123 Andria, Italy; 3 Andrology Unit, Department of Urology, University of Foggia, 71122 Foggia, Italy; 4 Department of Pathology, University of Foggia, 71122 Foggia, Italy; 5 Department of Radiology, University of Foggia, 71122 Foggia, Italy; 6 Department of Molecular Medicine and Surgery, (Solna), Karolinska Institutet, 17177 Stockholm, Sweden

**Keywords:** prostate cancer, standard biopsy, target biopsy, MRI fusion biopsy, prostate MRI

## Abstract

**Background:** The aim of this study was to validate externally a nomogram that relies on MRI volumetric parameters and clinical data to determine the need for a standard biopsy in addition to a target biopsy for men with suspicious prostate MRI findings. **Methods:** We conducted a retrospective analysis of a prospectively maintained database of 469 biopsy-naïve men who underwent prostate biopsies. These biopsies were guided by pre-biopsy multiparametric Magnetic Resonance Imaging (mpMRI) and were performed at two different institutions. We included men with a PIRADSsv 2.1 score from 3 to 5. Each patient underwent both an MRI–ultrasound fusion biopsy of identified MRI-suspicious lesions and a systematic biopsy according to our protocol. The lesion volume percentage was determined as the proportion of cancer volume on MRI relative to the entire prostate volume. The study’s outcomes were iPCa (Gleason Grade Group 1) and csPCa (Gleason Grade Group > 1). We evaluated the model’s performance using AUC decision curve analyses and a systematic analysis of model-derived probability cut-offs in terms of the potential to avoid diagnosing iPCa and to accurately diagnose csPCa. **Results:** The nomogram includes age, PSA value, prostate volume, PIRADSsv 2.1 score, percentage of MRI-suspicious lesion volume, and lesion location. AUC was determined to be 0.73. By using various nomogram cut-off thresholds (ranging from 5% to 30%), it was observed that 19% to 58% of men could potentially avoid undergoing standard biopsies. In this scenario, the model might miss 0% to 10% of diagnosis of csPCa and could prevent identifying 6% to 31% of iPCa cases. These results are in line with findings from the multi-institutional external validation study based on the IMPROD trial (n = 122) and the MULTI-IMPROD trial (n = 262). According to DCA, the use of this nomogram led to an increased overall net clinical benefit when the threshold probability exceeded 10%. **Conclusions:** This study supports the potential value of a model relying on MRI volumetric measurements for selecting individuals with clinical suspicion of prostate cancer who would benefit from undergoing a standard biopsy in addition to a targeted biopsy.

## 1. Introduction

In recent years, Magnetic Resonance Imaging (MRI) and MRI-targeted biopsy (TBx) have been proven to outperform systematic biopsy (SBx) alone for the diagnosis of clinically significant prostate cancer (csPCa) [1,2,3].

Specifically, these trials (PROMOD, PRECISION, MRI-FIRST) showed that the occurrence of high-grade group (GG) disease is uncommon in men whose MRI results are negative, demonstrating that TBx helps in detecting high-GG tumors that SBx fails to identify.

That explains the growing interest in evaluating if SBx is still needed in addition to TBx in men with positive MRIs. Avoiding SBx would reduce the risks of infection, bleeding, and pain associated with additional core sampling [4,5] but more importantly, would further reduce the incidental detection of clinically insignificant PCa (iPCa) [6]. Finally, the workload for pathologists evaluating biopsy cores would be reduced from reviewing 12–18 cores to 2–6 cores. Still, the risk of missing clinically significant PCa (csPCa) must be addressed, and the available evidence is conflicting.

Indeed, a recent multicenter study including 640 consecutive patients from 11 UK institutions has found that the clinical value of performing additional extensive unguided biopsies of nonsuspicious areas is limited and can often result in a diagnosis of insignificant cancers that do not need treatment [7]. Conversely, most of the documented series indicate that when performing only TBx, approximately 9% to 15% of csPCa can go undetected both in biopsy-naïve patients and in the repeat-biopsy setting [8,9], whereas a combined approach has shown a higher detection rate (DR) [10].

Several multivariable models to predict negative SBx have been developed to avoid systematic cores at the time of target biopsy [11,12]. None of these models showed excellent accuracy, and their clinical benefit is questionable. There are at least three reasons behind TBx failure: (1) misdiagnosis of the lesion by the radiologist either due to low quality of prostate MRI or misinterpretation; (2) presence of csPCa that is not visible on MRI; and (3) targeting error.

Falagario et al. developed a risk calculator for deciding when SBx should be performed in addition to TBx in med with suspicious MRI findings using the IMPROD trial development cohort and then they validated it using the MULTI-IMPROD validation cohort.

The developed nomogram included age, PSA, prostate volume, MRI-suspicion score (PIRADSv 2.1 score), MRI-suspicion lesion volume percentage, and lesion location and proved to be useful in reducing SBx at the time of positive MRI [13]. However, the development and validation cohort had centrally reviewed MRIs and unified MRI protocols [14]. The objective of this study was to externally validate the nomogram within the routine clinical setting of two tertiary care cancer centers. This validation involved utilizing biopsy data from men with MRI scans obtained from various institutions, each following different protocols.

## 2. Materials and Methods

### 2.1. Study Design and Population

We retrospectively analyzed data from prospectively maintained databases of men undergoing prostate biopsy at two institutions (the Department of Urology and Organ Transplantation at the University of Foggia, Italy and the Department of Urology at Bonomo Teaching Hospital, Andria, Italy). All patients underwent prebiopsy multiparametric MRI. Patients with negative MRI (PIRADS sv2.1 score of 1–2) were excluded from the analyses (n = 149). The final population consisted of 469 patients. All the study biopsies were taken from January 2014 to January 2022.

### 2.2. Multiparametric MRI

Multiparametric MRI examinations were performed in 11 different institutions (data about institutions are reported in Appendix A). All MRI protocols were compliant with Prostate Reporting and Data System (PIRADS) recommendations available at the time of diagnosis [15]. MRI at our institution, Policlinico Riuniti di Foggia, (n = 121) was performed using a 1.5 Tesla MR scanner (Achieva, Philips Healthcare, Best, the Netherlands) with a surface array coils (SENSE Flex surface). The mpMRI protocol consisted of A) 3-planes T2-weighed images; B) T1-weighed images in the axial plane; C) diffusion-weighted images in the axial plane at b-values ranging from 0 to 1500 s/mm^2^); and D) dynamic contrast-enhanced prostate MRI (DCE) performed using a T1-weighted high-resolution isotropic volume examination (THRIVE) on the axial plane with injection of 0.1 mL/kg of gadobutrol. Concerning other institutions, we do not have all the information required to describe their MRI protocols. However, MRIs were always performed using a 1.5 Tesla MR scanner and we always checked the provided sequences including 3 axial T2W images and DWI images using at least b values above 1500 and below 500.

Volumetric analysis was performed manually delineating whole prostate volumes and all MRI suspicious lesions on axial T2-weighted images.

Volumes were calculated as the sum of the prostate/lesion area on each slice multiplied by slice thicknesses (3mm). Cancer volume on MRI was calculated as the sum of all the lesion volumes in each patient. The total percentage of cancer on MRI was calculated as the ratio of cancer volume on MRI divided by the whole prostate volume. The index lesion (defined as the biggest lesion with the highest PIRADS score) location was considered in the model.

### 2.3. Biopsy Procedure and Histopathological Analysis

All patients underwent MRI-TRUS fusion biopsy of each MRI-suspicious lesion (three targeted biopsy cores) in addition to a fourteen-core systematic biopsy according to our standard biopsy template. Our standard template provides for seven cores per lobe: 2 peripheral cores (base and apex), 3 mid-gland cores, and 2 para-urethral cores (base and apex). All the biopsies were performed transrectally under TRUS guidance (BK Medical Flex Focus 500) using an 18 gauge/25 cm biopsy needle. Target cores were taken using one of two electromagnetic-tracked MRI/US fusion systems (Navigo, UC-CARE, Yokneam, ISR; bkFusion, GE Healthcare, Chicago, IL, USA).

Histological analysis of biopsy specimens was carried out by two experienced genitourinary pathologists using the 2014 International Society of Urological Pathology modified Gleason grading system [16]. Findings from MRI and clinical data were available to the pathologists at the time of microscopic evaluations.

### 2.4. Outcomes Measurements and Statistical Analysis

The primary outcome of this study was the detection rate of csPC in SBx, TBx, and overall (SBx + TBx), while secondary outcomes were the rate of iPCa avoidance and the number of SBx avoided.

The predicted probability of csPC in SBx was computed using the Logit formula and published coefficients [13]. No recalibration was performed.

The external validation was performed in three steps: discrimination was assessed by computing the area under the receiver operating characteristic curve (AUC) of the model, calibration was graphically tested by plotting observed vs. actual rates of csPCa on systematic biopsies, the clinical utility of the model was assessed using decision curve analysis (DCA). DCA was performed evaluating the net benefit of using the model with each threshold probability and was compared to the net benefit of using a strategy of systematic cores in everyone (red line) and a strategy of systematic cores in no one (blue horizontal line).

To provide physicians and patients with an outline for deciding when to perform SBx in addition to TBx, we performed a systematic analysis of model-derived cutoffs. Statistical analyses were performed using Stata-SE 16 (StataCorp LP, College Station, TX, USA). All tests were 2-sided with a significance level set at *p* < 0.05.

## 3. Results

The final population included 496 biopsy-naïve men. The clinical characteristics of the study population are presented in Table 1. At the time of biopsy, the median age of the patients was 67 (interquartile range (IQR): 61, 72) years. The median PSA value was 6.0 (IQR: 4.4, 9.0) ng/mL, and the median prostate volume was 50 (IQR: 39, 70) mL with a median PSA density of 0.13 (IQR: 0.08, 0.18).

Additionally, 48.6% of patients had a clinical suspicion of PCa at digital rectal examination (DRE).

A total of 149 (31.8%), 227 (48.4%), and 93 (19.8%) patients had PIRADS 3, 4, and 5 lesions, respectively. Index lesions were located in 349 (74.4%) patients in the peripheral zone.

Overall, 105 (22.4%) and 142 (30.3%) patients were diagnosed with iPCa and CsPCa respectively. CsPCa detection rates according to sampling methods were 26.7% (n = 125) in target cores and 23.5% (n = 110) in standard cores.

Concordance between the results of the two sampling methods (based on GGG of TBx only and SBx only) is presented in Table 2. Eighteen (18/469, 3.8%) patients had csPCa only on systematic cores, while 33 patients (33/469, 7.0%) had csPCa in target cores with negative results or iPCa at standard biopsy. Finally, out of the 105 patients diagnosed with iPCa, 44 (44/105, 41.9%) patients had iPCa on systematic cores only, 16 patients (16/105, 15.2%) had iPCa on target cores only and 45 (45/105, 42.9%) had iPCa in both the sampling methods.

### External Validation of the Model

The developed nomogram included age, PSA, prostate volume, MRI suspicion score (PIRADSv 2.1 score), MRI-suspicion lesion volume percentage, and lesion location.

The model predicts the probability of finding csPCa in the standard biopsy.

**Figure 1:**
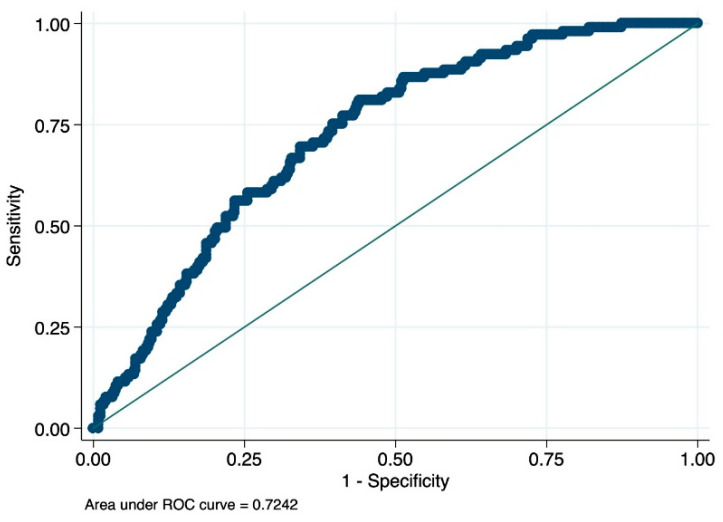
Area under the receiver operating characteristic (ROC) curve.

The AUC of the model was 0.72 (Figure 1).

**Figure 2:**
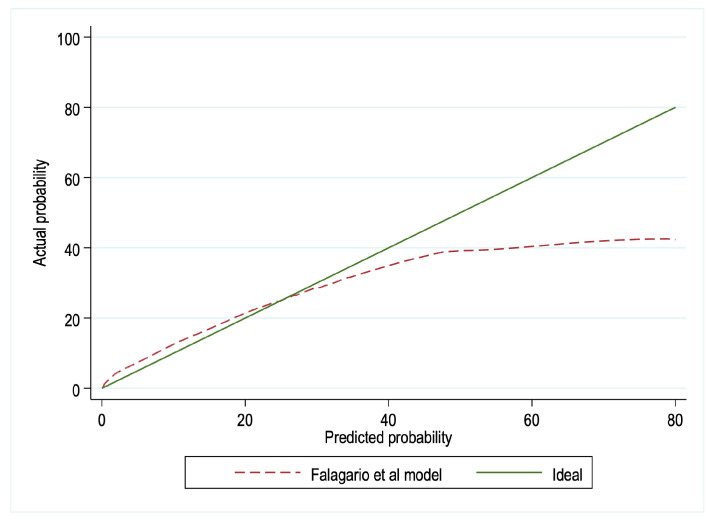
Calibration plot of observed vs. predicted probability of clinically significant prostate cancer in systematic biopsies.

We found that model-derived probabilities above 40% underestimated the actual probability of csPCa on SBx. On the contrary, the model’s calibration was excellent for probabilities below 40% (Figure 2). Consequently, recalibration was not deemed necessary, since the model’s primary clinical utility lies in the lowest range of probabilities. Within this range, clinicians may face uncertainty when deciding whether to proceed with SBx.

**Figure 3:**
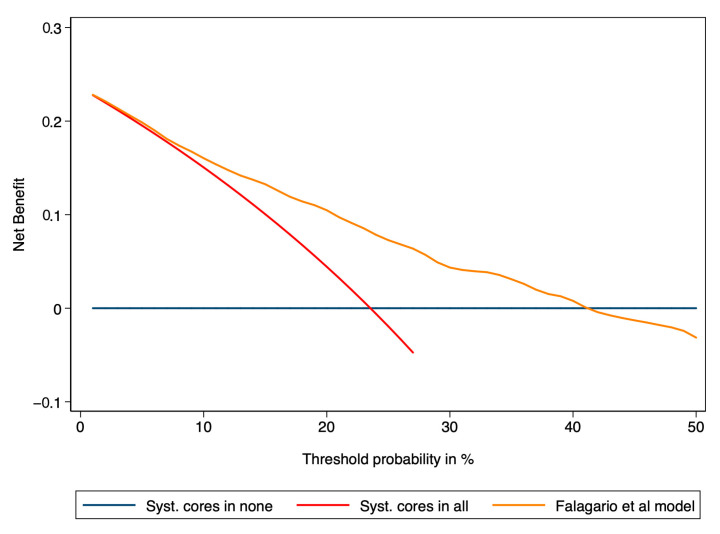
Decision curve analysis demonstrating net benefit between the threshold probabilities of 10% and 40% for the model predicting clinically significant prostate cancer in systematic biopsies. The model was compared to a strategy of systematic cores in everyone (red line) and a strategy of systematic cores in no one (blue horizontal line).

DCA showed an increased net clinical benefit compared to the net benefit of using a strategy of systematic cores in everyone and a strategy of systematic cores in no when the threshold probability was between 10% and 40% (Figure 3).

Finally, the systematic analysis of the model-derived cutoffs (Table 3) revealed that 19–58% of men would have avoided SBx while missing 0–10% of csPCA and avoiding the detection of 6–31% of iPCa. These results are similar to those found in the multi-institutional external validation study based on the IMPROD trial (n = 122) and the MULTI-IMPROD trial (n = 262).

## 4. Discussion

EAU Guidelines on PCa already recommend performing only TBx when MRI is positive in patients with prior negative biopsy, but not in biopsy naïve patients [17].

In this study, using data from a prospectively maintained database, we validated a model based on MpMRI volumetric parameters and clinical information that can help the physicians understand when SBx is really needed in biopsy naïve men who underwent TBx.

Our primary outcome was the detection rate of csPCa in SBx, TBx, and overall (SBx + TBx).

Some retrospective studies were carried out to assess if standard biopsy still has a role in detecting PCa or csPCa in biopsy-naïve patients with positive multiparametric Magnetic Resonance Imaging. These studies concluded that the addition of SBx to TBx did not result in a significant increase in the detection rate [7].

Recently, Porpiglia et al. published a prospective randomized control trial comparing TBx alone vs. a combined approach with TBx and SBx in biopsy-naïve patients [18].

The authors found that in patients who had not undergone prior biopsies, the TBx alone approach missed approximately 7.3% of PCa cases compared to the combined approach of TBx and SBx (TBx + SBx). However, when it came to the detection rate (DR) of csPCa, no notable differences were observed between performing TBx alone versus TBx + SBx [18].

Moreover, they noted that 10.3% of PCa cases and 7.2% of csPCa cases were exclusively diagnosed through SB despite positive mpMRI and negative target biopsy results.

Our findings align with this observation, as we found out that 7.7% of patients were diagnosed with csPCa exclusively on systematic cores.

On the other hand, we also found that 9.4% of patients had GGG1 diagnosis on target cores. Realistically, this could depend on the misreading of MRI by inexperienced radiologists and/or urologists but, actually, these results may potentially have a clinical importance as a recent study showed that Gleason 3 + 3 tumors that are visible on MRI have a higher risk of progressing than MRI-negative cancers [19].

One of the secondary outcomes of this study was the rate of iPCa avoidance which is of pivotal importance when it comes to population-based screening for PCa.

In 2022 Hugosson et al. from Gothenburg University designed the randomized GÖTEBORG-2 trial with the aim of assessing whether a screening algorithm including PSA testing followed by targeted biopsy only in patients with positive MRI results, would lead to reduced cases of iPCa overdiagnosis compared to current screening recommendations [20]. In this trial, they demonstrated that introducing prebiopsy MRI for all individuals with elevated PSA levels and performing only targeted biopsy for lesions with a PI-RADS score of 3 to 5 while avoiding biopsy for lesions with PI-RADS scores of 1 to 2, led to a 50% reduction in the detection of Gleason 3 + 3 cancers. However, this approach may slightly delay the detection of intermediate-risk tumors in a small fraction of patients.

Indeed, in the MRI-FIRST trial authors suggested that using mpMRI as a screening test for prostate cancer could be a valid approach but only for the diagnosis of highly aggressive tumors. Rouvière et al., in fact, reported that the added value of systematic biopsy was marginal for ISUP ≥ 3 tumors but not for ISUP 2 tumors as the detection of such tumors is improved when SBx and TBx are combined [3].

Even if some Gleason 3 + 4 disease is missed by a TBx–alone strategy as ‘‘MRI-invisible cancer’’, there are data showing that men with nonvisible Gleason 3 + 4 disease have an overall survival comparable with GGG 1 disease and that only men with visible ISUP 2 cancer experience worse outcomes [21,22].

In this scenario, the question remains whether some patients with low risk of csPCa but a positive MRI may avoid SBx.

In the MRI-FIRST trial authors found that the percentage of csPCa detected through targeted biopsy was significantly higher in lesions larger than 15 mm compared to lesions equal to or smaller than 15 mm. This difference can potentially be explained both by high-grade cancers tending to be larger than benign or low-grade lesions and small high-grade cancers tending to be missed by targeted biopsy [3].

Likewise, Porpiglia et al.’s model revealed that patients with negative TBx and a lesion diameter smaller than 10 mm are at high risk of missed PCa and they suggested considering these patients for additional SBx [18].

In this setting, the nomogram we validated has the potential to assist the physician in assessing the safety of avoiding SBx. The selection of a cutoff point is left to the physician’s judgment, as it depends on the number of PCa they are willing to potentially miss.

As an example, if a nomogram cutoff of 7.5% is chosen, there is a potential risk of missing 1.4% of csPCa while detecting only 10% of iPCa. Choosing a cutoff of 30% the risk of missing csPCa rises to 9.9% (Table 3). If the nomogram results of 40% or more the physician must perform SBx in addition to TBx because above 40% the model is no longer valid.

Despite the previously mentioned articles stating that TBx helps in detecting high-GG tumors that SBx fails to identify [1,2,3], a very recent study highlighted a very interesting issue about the oncological risk equivalence of positive target and systematic biopsy, supporting somehow the need of concomitant standard biopsy [23]. Gaffney et al. retrospectively analyzed data from 991 patients who had both systematic and MRI-targeted biopsy before undergoing radical prostatectomy and showed that patients with higher GGG on TBx compared to SBx did not have the same level of oncologic risk as those with identical GGG on both biopsy methods. They reported that when the grade is discordant between systematic and targeted biopsy, the risk is intermediate between the two grades, where lower GGG on targeted biopsy corresponds with decreased risk.

Beyond oncological safety, several additional factors are often mentioned as reasons to include systematic biopsy in treatment decision planning, including the assessment of focal therapy feasibility and the determination of nerve-sparing options for radical prostatectomy.

It has been demonstrated that incorporating dedicated uro-radiology MRI planning meetings prior to radical prostatectomy can enhance patients’ functional outcomes without requiring the inclusion of systematic biopsy [24].

As for focal therapies, a retrospective analysis conducted by Lee et al. revealed that nearly half of the men would have their treatment plans modified if a full systematic biopsy was performed instead of solely relying on a targeted biopsy, as it is crucial to accurately identify all tumor foci to ensure optimal patient selection and effective ablation of all regions containing csPCa [25].

A strength of our study is that we validated this nomogram using a cohort of patients from two referral centers performing biopsies on patients coming from different hospitals in the surrounding areas. Even if MRI images are always re-evaluated by the urologist performing the biopsy, we deal with MRI images performed and reported in centers with limited clinical expertise. This may explain our low detection rate of csPCa (26.7% in targeted cores and 23.5% in systematic cores), which is lower than expected, based on recent studies about the superiority of TBx. In the PRECISION trial, they reported a detection rate of csPCa of 38% in the MRI-targeted biopsy group and 26% in the standard biopsy group. In this trial, multiparametric prostate MRIs were performed by radiologists with an average experience of 5 years and an average number of prostate MRIs reported per year of 300, which is not our case [2]. Similarly, the detection rate of csPCa in the development cohort was higher (49.2% in standard cores and 60.7% in target cores) and the authors reported as a limitation to the validity of their findings that MRI and biopsies were performed according to the highest standard of care reflecting the daily clinical practice only in referral academic center [13].

Hence, we believe that the heterogeneity of our data better performs in an external validation study, as they more accurately mirror the practical clinical scenarios seen in non-academic settings, where radiologists and urologists are still in the early stages of their learning curve.

Despite this, the study is limited by its retrospective nature and by the lack of data on histopathological findings at radical prostatectomy. Future studies may also evaluate follow-up outcomes in those patients diagnosed with PCa on SBx or TBx alone.

To evaluate the practical applicability of the nomogram in clinical practice, prospective clinical trials will be necessary.

## 5. Conclusions

Considering the wide variability in the expertise of radiologists and urologists reading MRIs and performing biopsies and considering the importance of thoroughly mapping the prostate (to nerve-sparing and focal therapies decision making), currently, the standard biopsy is still necessary. However, the present study supports the potential utility of the improved predictive model in a particular subset of patients who would not benefit from Sbx in addition to TBx to reduce the risk of iPC overdiagnosis.

## Figures and Tables

**Table 1:** Baseline population characteristics, biopsy, and MRI Results.

	Overall Cohort N = 469
Age (yr)	67 (61, 72)
Previous Biopsy, n (%)	
None	469 (100%)
DRE, n (%)	
Negative	241 (51.4%)
Suspicious	228 (48.6%)
PSA, ng/ml	6.0 (4.4, 9.0)
Prostate volume, ml	50 (39, 70)
PSA Density	0.13 (0.08, 0.18)
PIRADS, n (%)	
3	149 (31.8%)
4	227 (48.4%)
5	93 (19.8%)
Index Lesion Location, n (%)	
PZ	349 (74.4%)
TZ-CZ	120 (25.6%)
Index Lesion Volume	0.52 (0.27, 1.44)
Biopsy Results, n (%)	
Negative	222 (47.3%)
GGG 1	105 (22.4%)
GGG 2	57 (12.2%)
GGG 3	47 (10.0%)
GGG 4	23 (4.9%)
GGG 5	15 (3.2%)
csPCa in Systematic Cores, n (%)	110 (23.5%)
csPCa in Target Cores, n (%)	125 (26.7%)

DRE: digital rectal examination; PZ: peripheral zone; TZ-CZ: transition zone, central zone; GGG: Gleason Grade Group; csPCa: clinically significant prostate cancer.

**Table 2:** Concordance between results of TBx only and SBx only.

		Systematic Cores		
		Negative	iPCa	csPCa
Target cores	Negative	221 (84.0%)	44 (45.8%)	11 (10.0%)
	iPCa	16 (6.1%)	45 (46.9%)	7 (6.4%)
	csPCa	26 (9.9%)	7 (7.3%)	92 (83.6%)

csPCa: clinically significant prostate cancer; iPCa: clinically insignificant prostate cancer.

**Table 3:** Systematic analysis of the model-derived cutoffs and respective estimated numbers of missed csPCa and avoided iPCa.

Nomogram Calculated Probability, Cutoff (%)	Patients Resulting Below Cutoff		Negative or iPCa on SBx		csPCa on SBx		csPCa Missed		iPCa Not Detected	
	N	(%) ^a^	N	(%) ^b^	N	(%) ^c^	N	(%) ^d^	N	(%) ^e^
5	88	18.8	85	23.7	3	2.7	0	0.0	6	5.7
7.5	119	25.4	111	30.9	8	7.3	2	1.4	10	9.5
10	134	28.6	124	34.5	10	9.1	2	1.4	13	12.4
12.5	154	32.8	142	39.6	12	10.9	2	1.4	15	14.3
15	178	38.0	164	45.7	14	12.7	3	2.1	19	18.1
17.5	199	42.4	179	49.9	20	18.2	5	3.5	22	21.0
20	211	45.0	191	53.2	20	18.2	5	3.5	25	23.8
22.5	229	48.8	203	56.5	26	23.6	8	5.6	26	24.8
25	248	52.9	217	60.4	31	28.2	10	7.0	28	26.7
27.5	262	55.9	227	63.2	35	31.8	11	7.7	31	29.5
30	274	58.4	234	65.2	40	36.4	14	9.9	33	31.4

^a^: out of the total number of biopsies performed (n = 469); ^b^: out of the total number of negative or iPCa in SBx (n = 359); ^c^: out of the total number of csPCa in SBx (n = 110); ^d^: out of the total number of csPCa in any core (n = 142); ^e^: out of the total number of iPCa diagnosed (n = 105). csPCa: clinically significant prostate cancer; iPCa: clinically insignificant prostate cancer.

## Data Availability

The data presented in this study are available on request from the corresponding author. The data are not publicly available due to ethical restrictions.

## Appendix A

The 11 institutions involved in performing prostate MRIs are:

1. Department of Radiology, Policlinico Riuniti di Foggia;
2. Bonomo Teaching Hospital, Andria;
3. IRCCS “Casa Sollievo della Sofferenza”, San Giovanni Rotondo;
4. IRCCS CROB, Rionero in Vulture;
5. Azienda Ospedaliero Universitaria Consorziale Policlinico di Bari;
6. Ospedale Generale Regionale “F.Miulli”, Acquaviva delle Fonti;
7. Ospedale “San Timoteo”, Termoli;
8. Centro Radiologico Potito, Campobasso;
9. Centro Radiologico Di Giovine-Virgantino, Lucera;
10. Centro Radiologico Di Giovine-Virgantino, San Severo;
11. Ospedale “G.Tatarella”, Cerignola.
